# Nitrate-Dependent Iron Oxidation: A Potential Mars Metabolism

**DOI:** 10.3389/fmicb.2018.00513

**Published:** 2018-03-20

**Authors:** Alex Price, Victoria K. Pearson, Susanne P. Schwenzer, Jennyfer Miot, Karen Olsson-Francis

**Affiliations:** ^1^Faculty of Science, Technology, Engineering and Mathematics, The Open University, Milton Keynes, United Kingdom; ^2^CNRS, Institut de Minéralogie, de Physique des Matériaux et de Cosmochimie, Muséum National d’Histoire Naturelle, Université Pierre et Marie Curie – Sorbonne Universités, UMR 7590, Paris, France

**Keywords:** iron, nitrate, Mars, astrobiology, chemolithotrophy, NDFO, nitrate-dependent ferrous iron oxidation, anaerobic

## Abstract

This work considers the hypothetical viability of microbial nitrate-dependent Fe^2+^ oxidation (NDFO) for supporting simple life in the context of the early Mars environment. This draws on knowledge built up over several decades of remote and *in situ* observation, as well as recent discoveries that have shaped current understanding of early Mars. Our current understanding is that certain early martian environments fulfill several of the key requirements for microbes with NDFO metabolism. First, abundant Fe^2+^ has been identified on Mars and provides evidence of an accessible electron donor; evidence of anoxia suggests that abiotic Fe^2+^ oxidation by molecular oxygen would not have interfered and competed with microbial iron metabolism in these environments. Second, nitrate, which can be used by some iron oxidizing microorganisms as an electron acceptor, has also been confirmed in modern aeolian and ancient sediment deposits on Mars. In addition to redox substrates, reservoirs of both organic and inorganic carbon are available for biosynthesis, and geochemical evidence suggests that lacustrine systems during the hydrologically active Noachian period (4.1–3.7 Ga) match the circumneutral pH requirements of nitrate-dependent iron-oxidizing microorganisms. As well as potentially acting as a primary producer in early martian lakes and fluvial systems, the light-independent nature of NDFO suggests that such microbes could have persisted in sub-surface aquifers long after the desiccation of the surface, provided that adequate carbon and nitrates sources were prevalent. Traces of NDFO microorganisms may be preserved in the rock record by biomineralization and cellular encrustation in zones of high Fe^2+^ concentrations. These processes could produce morphological biosignatures, preserve distinctive Fe-isotope variation patterns, and enhance preservation of biological organic compounds. Such biosignatures could be detectable by future missions to Mars with appropriate instrumentation.

## Introduction

Mars, the red planet, has inspired the search for extraterrestrial life since the early days of the telescope, and continues to do so with perceptions of its habitability—or even inhabitation—changing with advances in exploration capabilities and knowledge of martian environments from images and data (Filiberto and Schwenzer, 2017). The present-day surface of Mars is cold, dry, and exposed to ionizing and UV radiation, conditions deemed detrimental to life, but evidence in the geological and geomorphological record of Mars confirms warmer, wetter, and potentially more favorable surface conditions during the Noachian period of early Mars (4.1–3.7 Ga) (Carr and Head, 2010). During this period, evidence for a denser atmosphere and less oxidizing conditions suggests that more hospitable surface environments for life may have prevailed (Carr and Head, 2010; Mangold et al., 2012), including: large-scale fluvial systems (Malin and Edgett, 2003; Irwin et al., 2005; Fassett and Head, 2008; Mangold et al., 2012; Williams et al., 2013), lacustrine environments (Grotzinger et al., 2014; Rampe et al., 2017a), and impact-generated hydrothermal systems (Schwenzer and Kring, 2009; Osinski et al., 2013). Evidence for these environments comes from lake bed sediments, such as those identified at Gale Crater, which the NASA Mars Science Laboratory rover (Curiosity) is investigating in detail (e.g., Grotzinger et al., 2015). Phyllosilicates and other hydrated minerals have also been observed from orbit (Gendrin et al., 2005; Bibring et al., 2006; Chevrier et al., 2007) and from the ground (Squyres et al., 2004; Ehlmann et al., 2011). In light of our developing understanding of Mars as a dynamic planet with a complex history, this review appraises the viability of microbial nitrate-dependent iron oxidation as a candidate metabolism with regard to past and present martian environments.

## Mars – Geological Background

For a better understanding of the contrast between the detrimental conditions on the surface of present-day Mars and the wetter, more clement past of martian surface environments, two specific potentially habitable environments are discussed here: (1) the ancient lake bed investigated by the Curiosity rover at Gale Crater (Grotzinger et al., 2014, 2015; Palucis et al., 2016) and (2) the impact-generated hydrothermal environment discovered in the rim of Endeavour Crater by the MER Opportunity rover (Squyres et al., 2012; Arvidson et al., 2014; Fox et al., 2016).

The ancient lake bed at Gale Crater is likely to be one of many that formed within impact craters on Mars (Cabrol and Grin, 1999). Conglomerates, cross-bedded sandstones, siltstones, and mudstones have been identified by the Curiosity rover, allowing for a detailed understanding of water flow, standing water conditions, and even temporary periods of desiccation (Vaniman et al., 2013; Williams et al., 2013; Grotzinger et al., 2014, 2015; Palucis et al., 2016; Hurowitz et al., 2017). The mineralogy and geochemistry of Gale Crater sediments suggest that the conditions in this ancient lake were temperate and pH-neutral, suitable for the maintenance of life for most of the time (Grotzinger et al., 2014, 2015), although excursions to, or local areas of, acidic conditions are evidenced by the discovery of jarosite (Rampe et al., 2017a,b). Post-depositional diagenetic and alteration processes, such as the dissolution of primary minerals, the formation of calcium-sulfate veins, cementation, desiccation, or even changes to the chemistry of the incoming sediment load due to external silicic volcanism, will have changed the environmental conditions multiple times, leading to a complex association of environmental conditions variable in space and time (Bridges et al., 2015; Johnson et al., 2016; Schwenzer et al., 2016; Frydenvang et al., 2017; Nachon et al., 2017; Rampe et al., 2017a; Yen et al., 2017). Further, Gale Crater sediments are reported to contain bioessential elements such as hydrogen, phosphorus, oxygen, and nitrogen, variable iron and sulfur oxidation states as possible energy sources, and perhaps even complex organic molecules at concentrations that could have supported past life (Vaniman et al., 2013; Grotzinger et al., 2014; Stern et al., 2015; Morris et al., 2016; Sutter et al., 2016).

Orbital observations have shown that many craters bear evidence of impact-generated hydrothermal activity (Marzo et al., 2010; Mangold et al., 2012), and ground-based exploration by the MER rover Opportunity revealed an impact-generated hydrothermal system at Endeavour Crater (Squyres et al., 2012; Arvidson et al., 2014; Fox et al., 2016). Characteristic products of such alteration are clay minerals, with the most complete succession of minerals ascribed to impact-generated hydrothermal activity found in the nakhlite meteorites (Changela and Bridges, 2010; Bridges and Schwenzer, 2012; Hicks et al., 2014). While these meteorites have an unknown geological context, and thus the impact-generated nature of the alteration remains an informed guess, the opportunity to investigate the succession of minerals with Earth-based instrumentation adds significant detail to an understanding of the compositional, reduction–oxidation (redox), and pH evolution of such alteration processes. For example, the alteration reactions evident in the nakhlites indicate a change in the redox conditions from Fe^2+^ precipitates to Fe^3+^ precipitates in the course of the formation of the assemblage (Bridges and Schwenzer, 2012; Hicks et al., 2014). Investigating such details is, to date, beyond the capability of rovers and landers, but provides essential information for assessing the habitability of the site during and after the hydrothermal activity.

Active terrestrial hydrothermal systems observed today are linked to active tectonic processes or volcanism, which drive water circulation on present-day Earth; there is no evidence of a sufficiently large or sufficiently young crater in which an active impact-generated hydrothermal system could exist. However, evidence for past hydrothermal systems is observed in the form of hydrothermal mineral veins around many terrestrial craters, e.g., Chicxulub, Manicouagan, Sudbury, and many others (see Pirajno, 2009; Osinski et al., 2013 for reviews). The difference between impact-generated and volcanic hydrothermal systems is the addition of species from degassing magma in the latter system, mainly HCl, H_2_HSO_4_, and other volatiles (Pirajno, 2009; Osinski et al., 2013), though fluids in both types of systems dissolve the wall rock and deposit secondary phases as conditions change throughout their lifetime. In both cases, the hydrothermal systems contain abundant bioessential elements (carbon, hydrogen, oxygen, nitrogen, and sulfur) that support diverse microbial communities (Arnold and Sheppard, 1981; Welhan and Craig, 1983; Charlou and Donval, 1993; Wheat et al., 1996; Konn et al., 2009). On Mars, hydrothermal systems caused by large hypervelocity impacts could provide warm water conditions even in periods of cold climate. With estimated life-times of 150–200k years even for modest craters (100–180 km diameter) the size of Gale, and with cycles of continuous mineral dissolution and precipitation maintaining the availability of redox substrates during that time, impact-generated hydrothermal systems could have provided localized hospitable zones (Abramov and Kring, 2005; Schwenzer and Kring, 2009).

These two examples of martian environments (lacustrine and impact-generated hydrothermal systems) demonstrate the diversity of potentially habitable environments (as we understand them today) on ancient Mars. In early surface environments, where the conditions were less inhospitable than the present-day, both phototrophic (solar energy-driven) and chemotrophic (chemical energy-driven) primary producers may have been viable, possibly producing enough organic carbon for the subsequent development of heterotrophy and a complex web of microbial life. As the environment evolved from “warm and wet” to “cold and dry,” life would have likely become limited to the sub-surface environment (Nixon et al., 2012), protected from the adverse surface conditions and, as such, may have become limited to light-independent chemolithotrophic (inorganic chemical energy-driven) metabolisms.

Laboratory-based Mars simulation experiments, using analog regolith or brine, and theoretical modeling have suggested that chemolithotrophic life could persist in the sub-surface martian environment across a wide range of pH, salinity, desiccation, and temperature (Parnell et al., 2004; Amils et al., 2007; Jepsen et al., 2007; Gronstal et al., 2009; Chastain and Kral, 2010; Smith, 2011; Popa et al., 2012; Hoehler and Jørgensen, 2013; Montoya et al., 2013; Summers, 2013; Bauermeister et al., 2014; Oren et al., 2014; King, 2015; Fox-Powell et al., 2016; Schuerger and Nicholson, 2016).

## Chemolithotrophy on Mars

Chemolithotrophic microorganisms harvest energy from redox reactions using inorganic substrates that are available in the environment. This metabolic strategy involves the transfer of electrons donated by the inorganic substrate, through the electron transport chain for ATP production, to a final acceptor. Chemolithotrophy is pivotal for biogeochemical cycling on Earth, such as iron, nitrogen, and sulfur cycling, and for rock weathering (Madigan et al., 2009).

**FIGURE 1:**
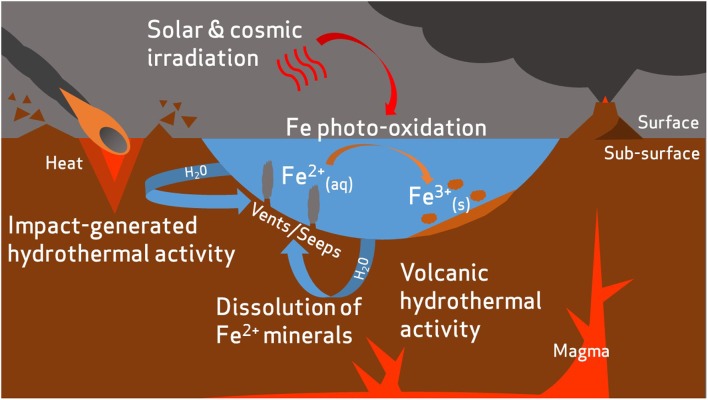
Hypothetical transport of iron on early Mars. Reduced iron is released into aqueous environments by dissolution of ferrous minerals. This process could be accelerated by volcanic or impact-generated hydrothermal activity (McSween et al., 2009). Some dissolved iron may be photo-oxidized by solar UV radiation to ferric compounds and deposited as sediments (Nie et al., 2017).

The iron-rich nature of Mars raises possibilities regarding the feasibility of iron biogeochemical cycling. Martian crustal geology is dominated by rocks of basaltic composition, which contain abundant FeO in quantities roughly twice those observed in comparable basalts on Earth (McSween et al., 2003, 2009). Though the planet’s surface is widely colored by iron oxides, reduced iron, Fe^2+^, exists as little as a few centimeters beneath the surface (Vaniman et al., 2013). Indeed, Fe^2+^-bearing minerals such as olivine [(Mg, Fe^2+^)_2_SiO_4_] have been detected across wide areas of the martian surface (Hoefen et al., 2003) and large amounts of basaltic glass (amorphous Fe^2+^-containing materials) are contained within martian crustal rocks (Morris et al., 2006a,b; McSween et al., 2009). An active hydrological cycle, combined with prevailing reducing conditions during the Noachian period, is likely to have facilitated large-scale transport of iron (**Figure 1**).

On early Earth, iron biogeochemical cycling and the occurrence of iron redox couples were crucial to the biosphere, to provide energy sources and because of the role of iron in many metalloproteins such as cytochromes, nitrogenases, and hydrogenases (Canfield et al., 2006; Hoppert, 2011; Raiswell and Canfield, 2012). Iron can act as either an electron acceptor or donor dependent on its redox state (Miot and Etique, 2016). Iron oxidizing microorganisms have been shown to utilize Fe^2+^ directly after its dissolution from minerals such as olivine (Santelli et al., 2001), and a similar process may have operated within potentially habitable environments on Mars. Conversely, microbial iron reduction commonly utilizes electrons donated from organic substrates, H_2_ or S^0^, with oxidized Fe^3+^ as the final electron acceptor (Lovley and Phillips, 1988; Lovley et al., 1989).

**FIGURE 2:**
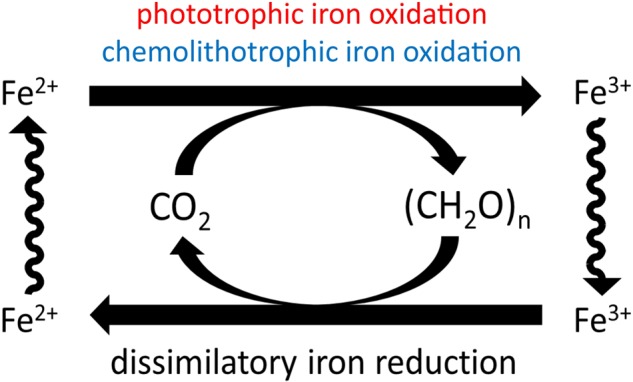
Hypothetical martian biogeochemical cycle of iron. Nealson (1997) suggested combination of phototrophic iron oxidation (Ehrenreich and Widdel, 1994) and heterotrophic iron reduction (Myers and Nealson, 1988) to give a hypothetical iron cycle. Carbon cycles are driven by solar and chemical energy sources. Iron is both the oxidant and the reductant for the cycle. Chemolithotrophic iron oxidation is proposed as an alternative to phototrophic iron oxidation, as the post-Noachian Mars surface environment may restrict opportunities for phototrophy, and any mechanism of iron oxidation in more recent periods may necessarily be light-independent.

A hypothetical ‘loop’ of biologically mediated martian iron cycling (**Figure 2**) was first proposed by Nealson (1997), which included both iron reduction and also phototrophic iron oxidation (Ehrenreich and Widdel, 1994); the plausibility of iron reduction has been appraised previously (Nixon et al., 2012, 2013; Nixon, 2014). However, Nealson’s model has limited applications to present-day Mars because of prohibitive conditions for phototrophic life in surface environments that prevent closure of this ‘loop’ for biogeochemical iron cycling.

Although research suggests that phototrophs may be sufficiently protected inside various micro-habitats within ice, halite, Fe^3+^-rich sediments, and impact-shocked rocks to withstand modern martian UV flux and remain photosynthetically productive (Cockell and Raven, 2004), the effect of desiccation, in combination with UV irradiation, would prevent dispersal and negatively impact viability (Cockell et al., 2005). Additionally, a lack of liquid water at the surface of Mars would be detrimental to life (Martín-Torres et al., 2015). A plausible alternative to a phototrophic iron oxidizer would be a chemolithotrophic iron oxidizer, which can obtain energy from redox reactions involving inorganic substances. This would allow for a light-independent iron cycle, which could have existed at the surface or in the sub-surface of early Mars and even continue today in deep sub-surface groundwaters (Michalski et al., 2013).

## Biotic Iron Oxidation

Abiotic Fe^2+^ oxidation occurs as a function of oxidant concentration, pH, temperature, and Fe^2+^ concentration (Ionescu et al., 2015). On Earth, low pH (<4) prevents the abiotic oxidation of Fe^2+^ by atmospheric O_2_, allowing biotic oxidation (using oxygen as the electron acceptor) to dominate (Morgan and Lahav, 2007). Evidence from evaporitic palaeoenvironments on Mars suggests historic low pH (<3.5) conditions existed in certain regions (Gendrin et al., 2005; Squyres and Knoll, 2005; Ming et al., 2006), although neutral–alkaline pH-associated clays are also observed in older terrains (Bibring et al., 2006). The transition to more arid conditions is thought to have coincided with a general shift from widespread clay formation to evaporitic sulfate precipitation at the surface (Bibring et al., 2006; Chevrier et al., 2007), resulting in increasingly acidic brines that may promote this form of biotic iron oxidation (Tosca and McLennan, 2006, 2009). However, given that only trace quantities (1450 ppm) of oxygen exist in the modern martian atmosphere (Mahaffy et al., 2013), aerobic, acidophilic iron oxidation is unlikely at the surface today (Bauermeister et al., 2014).

An alternative to aerobic iron oxidizers is microaerophilic neutrophilic iron oxidizers (NFeOs), which are able to compete with abiotic oxidation at near neutral pH. On Earth, this form of metabolism is largely restricted to oxic–anoxic boundary zones, where chemical oxidation is much slower (Roden et al., 2004). Phylogenetic studies have identified NFeOs in a variety of terrestrial environments including arctic tundra, Icelandic streams, deep-ocean vents, iron-rich soils, and temperate ground waters (Emerson and Moyer, 2002; Edwards et al., 2003; Emerson and Weiss, 2004; Cockell et al., 2011; Hedrich et al., 2011; Emerson et al., 2015). Many NFeOs are psychrophilic (Edwards et al., 2003, 2004), which could be linked to the much lower rate of abiotic iron oxidation at low temperatures (Millero et al., 1987).

On Mars, regions of higher partial pressure of oxygen in the modern sub-surface, relative to the surface, have been proposed as tolerable for microaerophiles today (Fisk and Giovannoni, 1999). King (2015) also argued that aerobic activity could be supported by the oxygen concentrations recorded by the Curiosity rover (Mahaffy et al., 2013); however, aerobic metabolism would be restricted, since oxygen diffusion distances in sediments are often limited to a few millimeters (Revsbech et al., 1980; Reimers et al., 1986; Visscher et al., 1991). Furthermore, there is evidence to suggest that redox stratification, seen in standing water bodies on Earth (Comeau et al., 2012), also occurred in martian lakes such as Gale Crater, resulting in an anoxic bottom layer (Hurowitz et al., 2017). Even assuming an oxygen-rich early martian atmosphere such as that suggested by Tuff et al. (2013), deeper waters, sediments, and the sub-surface would have been largely anoxic. As such, whatever the martian atmospheric oxygen concentration, potential habitats for anaerobically respiring light-independent chemolithotrophs would have been prevalent on ancient and present-day Mars.

Anaerobic chemotrophic iron oxidation is known to occur in terrestrial anoxic waters and sediments of approximately circumneutral pH (Straub et al., 1996; Benz et al., 1998; Kappler and Straub, 2005; Chakraborty and Picardal, 2013). Data from Curiosity at Gale Crater have shown that the Sheepbed mudstone formation at Yellowknife Bay contains abundant clay minerals, indicating a circumneutral pH environment during sedimentation (Vaniman et al., 2013; Grotzinger et al., 2014; Bridges et al., 2015; Schwenzer et al., 2016). The conditions associated with Gale Crater are not unique and can be inferred for other sites on Mars. For example, circumneutral aqueous alteration during both the Noachian and across the Noachian–Hesperian boundary has been proposed based on orbital data of Jezero crater (Ehlmann et al., 2008, 2009), indicating further environments in which anaerobic iron oxidation may have occurred.

## Availability of Electron Acceptors

In the absence of molecular oxygen, chemolithotrophic iron oxidizers would be limited by the availability of alternative electron acceptors, such as perchlorates and nitrates, for metabolic redox reactions (Straub et al., 1996; Benz et al., 1998; Kappler and Straub, 2005; Chakraborty and Picardal, 2013).

Studies at multiple locations on Mars have confirmed the presence of perchlorate (Hecht et al., 2009; Navarro-González et al., 2010; Glavin et al., 2013; Kounaves et al., 2014). Perchlorate-reducing bacteria, some able to grow at 0.4 M ${\mathrm{CIO}}_{4}^{-}$ (Oren et al., 2014)—concentrations exceeding those found on Mars (Stern et al., 2017)—have been isolated from terrestrial environments. Many are able to promote Fe^2+^ oxidation when perchlorate or nitrate is provided as an electron acceptor (Bruce et al., 1999; Chaudhuri et al., 2001; Lack et al., 2002), though energy conservation leading to growth is yet to be described in the case of perchlorate reduction coupled to Fe^2+^ oxidation.

Nitrate is thus a more feasible electron acceptor for martian iron oxidation, having been observed as the oxidant in iron-oxidizing metabolisms of growth-phase cultures (Hafenbradl et al., 1996; Straub et al., 1996; Benz et al., 1998; Straub and Buchholz-Cleven, 1998). However, until the recent discovery of nitrates on the surface of Mars (Stern et al., 2015), nitrate reducers have been largely overlooked with regard to Mars astrobiology. The following sections discuss the discovery of nitrates on Mars and the feasibility of nitrate-dependent iron oxidation as a plausible metabolism for now closing the biological iron ‘loop’ on Mars (**Figure 2**).

## Nitrates and Nitrogen Cycling on Mars

**FIGURE 3:**
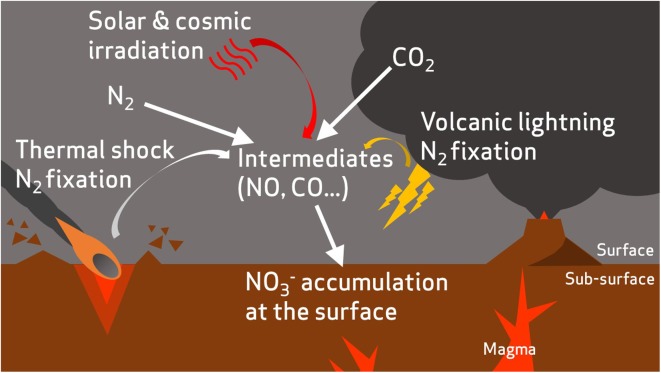
A hypothetical incomplete nitrogen cycle on early Mars. Atmospheric nitrogen is fixed to oxidized nitrogen species via abiotic processes such as volcanic lightning (Stern et al., 2015), thermal shock during impacts (Summers and Khare, 2007), and irradiation from solar and cosmic sources (Smith et al., 2014).

The geochemical evidence of nitrates on the surface of Mars comes from *in situ* analysis of mudstone at Gale Crater by Curiosity (Stern et al., 2015) and from analysis of the EETA79001 and Nakhla martian meteorites (Grady et al., 1995; Kounaves et al., 2014). It has been proposed that these nitrates may have formed through photochemical processing (Smith et al., 2014) of the low abundance molecular nitrogen (1.9%) in the martian atmosphere (Mahaffy et al., 2013), volcanic-induced lightning, or thermal shock from impacts (Stern et al., 2015), and may have resulted in large accumulated quantities of nitrates during the early history of the planet (Manning et al., 2009; Stern et al., 2017) (**Figure 3**).

Although it is not believed that nitrate deposition currently operates on the martian surface (Stern et al., 2015), interest in the martian nitrogen cycle has been reignited because of recent spacecraft observations of atmospheric nitrogen in the upper atmosphere (Stevens et al., 2015). On Earth, the production of molecular nitrogen is primarily facilitated by microbes through denitrification (Fowler et al., 2013). Biological denitrification on Mars could have contributed to an early nitrogen cycle during the Noachian period, although Mars’ atmosphere (including its primordial atmosphere) has long been suspected to have had a low nitrogen abundance relative to Earth (Fox, 1993). Nevertheless, the presence of nitrates as a plausible electron acceptor expands the range of microbial metabolisms that could be considered potentially viable on Mars. Of particular interest is the coupling of nitrate reduction to iron oxidation, which could exploit the vast martian reservoir of Fe^2+^ ions via nitrate-dependent Fe^2+^ oxidation (NDFO).

## Nitrate-Dependent Fe^2+^ Oxidation (NDFO)

Nitrate-dependent Fe^2+^ oxidation metabolism was identified on Earth two decades ago (Straub et al., 1996), yet the detailed biochemical mechanisms involved are still unresolved (e.g., Carlson et al., 2013). Early studies reported Fe^2+^ oxidation balanced with nitrate reduction in mixed cultures and isolates from anaerobic freshwater, brackish water, and marine sediments (Hafenbradl et al., 1996; Straub et al., 1996; Benz et al., 1998). There are only a few known isolates capable of this metabolism (see **Table 1**), but this is likely to be an under-representation of the true diversity and prevalence of these organisms (Straub and Buchholz-Cleven, 1998); NDFO may actually be an innate capability of all nitrate reducers (Carlson et al., 2013; Etique et al., 2014). Enzymatic Fe^2+^ oxidation by NDFO has never been proven and a detailed proteomic study of the NDFO species *Acidovorax ebreus* definitively demonstrated that this strain lacks any specific Fe^2+^ oxidoreductase (Carlson et al., 2013). Alternatively, electrons may transit from Fe^2+^ to other periplasmic enzymes (e.g., enzymes from the nitrate reduction chain) and abiotic side reactions between Fe^2+^ and reactive nitrogen species (NO and ${NO}_{2}^{-}$) produced upon nitrate reduction could also account for Fe^2+^ oxidation (Carlson et al., 2013; Klueglein et al., 2014, 2015).

**Table 1:** Examples of microbial species capable of nitrate-dependent iron oxidation.

Isolate	Respiration	e^-^ Donor	e^-^ Acceptor	Optimum pH	Optimum temperature (°C)	Metabolism	NDFO Growth	Reference
*Thiobacillus denitrificans*	Obligate anaerobe	S-species/Fe^2+^	${NO}_{3}^{-}$	6.90	30	Autotrophic	Unclear	Straub et al., 1996
*Pseudogulbenkiania* sp. strain 2002	Facultative aerobe	Fe^2+^	${NO}_{3}^{-}$	6.75–8.00	37	Autotrophic	Yes	Weber et al., 2006b
*Paracoccus* sp. strain KS1	Facultative aerobe	Organics/S/Fe^2+^	${NO}_{3}^{-}$	7.00	37	Heterotrophic	No	Kumaraswamy et al., 2006
*Acidovorax* sp. strain BoFeN1	Facultative anaerobe	Organics/Fe^2+^	${NO}_{3}^{-}$	6.80	30	Mixotrophic	Unclear	Kappler et al., 2005
*Ferroglobus placidus*	Obligate anaerobe	Fe^2+^/H_2_/S^2-^	${NO}_{3}^{-}$	7.00	85	Autotrophic	Yes	Hafenbradl et al., 1996
*Azospira* sp. strain PS	Facultative anaerobe	Fe^2+^/humic acids	${NO}_{3}^{-}$/${\mathrm{CIO}}_{4}^{-}$	7.00	26	Mixotrophic	No	Lack et al., 2002; Byrne-Bailey and Coates, 2012
*Acidovorax* sp. strain BrG1	Facultative anaerobe	Organics/Fe^2+^	${NO}_{3}^{-}$	6.70	28–35	Heterotrophic	No	Straub et al., 1996, 2004
*Aquabacterium* sp. strain BrG2	Facultative anaerobe	Organics/Fe^2+^	${NO}_{3}^{-}$	6.40–6.70	28	Heterotrophic	Unclear	Straub et al., 1996, 2004
*Thermomonas* sp. strain BrG3	Facultative anaerobe	Organics/Fe^2+^	${NO}_{3}^{-}$	6.70	32–35	Heterotrophic	No	Straub et al., 1996, 2004
*Klebsiella mobilis*	Facultative aerobe	Organics	${NO}_{3}^{-}$	7.00	30	Heterotrophic	No	Etique et al., 2014


Nitrate-dependent Fe^2+^ oxidation microorganisms have to balance (a) a potential energy gain from coupled iron oxidation and nitrate reduction and (b) energy consumption to overcome the toxicity of Fe^2+^ and reactive nitrogen species (Carlson et al., 2012, 2013). Although Fe^2+^ oxidation coupled to nitrate reduction to nitrite provides less energy (-481.15 kJ mol^-1^ ${NO}_{3}^{-}$) than both organotrophic denitrification (-556 kJ mol^-1^ ${NO}_{3}^{-}$) and organotrophic nitrate ammonification (-623 kJ mol^-1^ ${NO}_{3}^{-}$) (Strohm et al., 2007), this reaction is exergonic at circumneutral pH (-481.15 kJ mol^-1^ ${NO}_{3}^{-}$), and may theoretically provide enough energy to sustain growth under mixotrophic (Muehe et al., 2009; Weber et al., 2009) or autotrophic conditions (Laufer et al., 2016). At the same time, ferruginous conditions stimulate metal efflux pumping and stress response pathways (Carlson et al., 2013) and may thus impair the energetic budget of NDFO.

The terrestrial NDFO microbes currently described in the literature are phylogenetically diverse, including an archaeal species, as well as representatives of the alph-, beta-, gamma-, and delta-proteobacteria (Hafenbradl et al., 1996; Kappler et al., 2005; Kumaraswamy et al., 2006; Weber et al., 2009; Chakraborty et al., 2011). The isolation of a member of the euryarchaeota capable of NDFO from a submarine vent system (Hafenbradl et al., 1996) is suggestive that NDFO may have been a very early microbial process on Earth, due to the implication of such environments in the earliest evolution of life (Martin et al., 2008). Ilbert and Bonnefoy (2013) postulated that the mechanisms of biological anaerobic iron oxidation have arisen independently several times on Earth in an example of convergent evolution (i.e., similar strategies are adopted by genetically distant species). This widespread phylogeny, evidence from iron palaeochemistry, physiology, and redox protein cofactors involved in these pathways, suggests that NDFO may be the most ancient iron oxidation pathway in terrestrial life (Ilbert and Bonnefoy, 2013). Indeed, NDFO microbes have been implicated, alongside anoxygenic Fe^2+^-oxidizing phototrophy, in iron cycling and the production of early banded iron formations prior to the full oxygenation of the atmosphere on Earth (Weber et al., 2006a; Busigny et al., 2013; Ilbert and Bonnefoy, 2013). Thus, NDFO may be relevant to any putative early biosphere on Mars, where the conditions are favorable to this metabolism.

## Feasibility of NDFO on Early Mars

**FIGURE 4:**
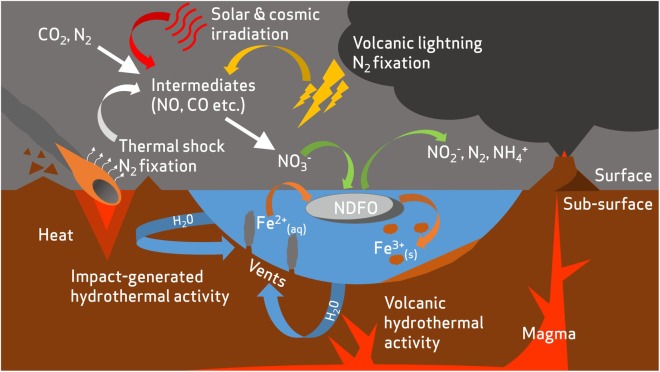
Overview of potential redox substrate sources for nitrate-dependent iron oxidizing microorganisms in the early Mars environment. Nitrates are produced from an early atmospheric nitrogen reservoir by fixation from volcanic lightning (Stern et al., 2015), thermal shock during impacts (Summers and Khare, 2007), and irradiation from solar and cosmic sources (Smith et al., 2014). Reduced iron is released into aqueous environments by mineral dissolution, a process accentuated by hydrothermal activity (Emerson and Moyer, 2002; McSween et al., 2009). A fuller description of abiotic nitrogen fixation pathways is available in Summers et al. (2012).

The relevance of NDFO as a plausible metabolism for putative life on Mars had, until recently, been overlooked due to the lack of evidence of nitrogen species on Mars, although the theoretical possibility of NDFO was explored using numerical modeling with hypothetical nitrate sources (Jepsen et al., 2007). The newly found availability of nitrates helps to close the ‘loop’ of potential chemotrophic iron cycling on Mars (**Figure 2**), since it could provide a ready source of electron acceptors for NDFO organisms (**Figure 4**); the concentration of nitrates detected at Gale Crater (Stern et al., 2015) is consistent with predictions of a 5 × 10^15^ mol global nitrate reservoir from past impact processing (Manning et al., 2009). It should be noted that the highest nitrate concentrations (1,100 ppm) determined by Curiosity were present in the sedimentary rocks with the least evidence of subsequent alteration, suggesting a period of more active nitrate production during sediment deposition, which was then followed by leaching of some sediments (Stern et al., 2015).

**FIGURE 5:**
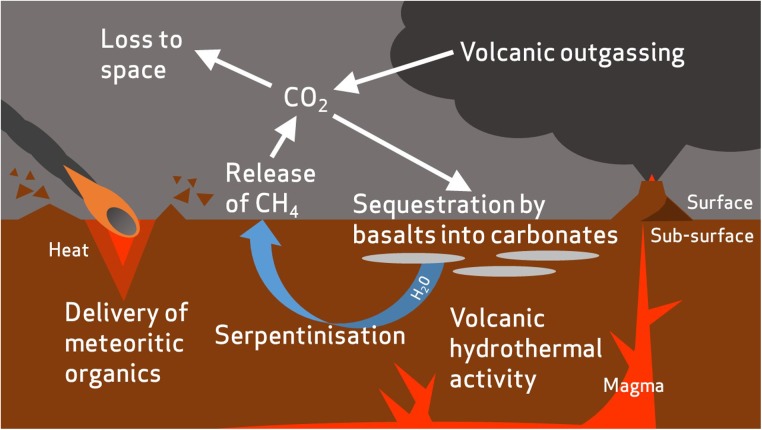
Summary of the proposed processes in carbon cycling on early Mars. Atmospheric carbon dioxide is sequestered by basalts to form carbonate minerals (Edwards and Ehlmann, 2015). The carbon is then remobilized by hydrothermal fluids and incorporated into simple organic compounds, such as methane, by serpentinization reactions (Chassefière and Leblanc, 2011). Carbon dioxide is gradually lost to space due to erosion of the atmosphere by solar winds. Meteorites are also likely to have delivered an inventory of organic carbon to the surface and sub-surface of Mars (Yen et al., 2006).

The modern martian atmosphere is 95.9% CO_2_ (Mahaffy et al., 2013), and CO_2_ is likely to have also formed a major proportion of the denser early Mars atmosphere (Ramirez et al., 2014; Jakosky et al., 2017) (**Figure 5**). Microbes that can utilize inorganic atmospheric carbon would therefore hold an advantage in the Mars environment. Although a low energy-yielding metabolism, a some species (*Pseudogulbenkiania* sp. strain 2002 and the hyperthermophilic archaeon *Ferroglobus placidus*) have been found to fix carbon autotrophically from CO_2_ and other inorganic sources during growth by NDFO (Hafenbradl et al., 1996; Weber et al., 2006b, 2009), providing an alternative carbon assimilatory capability relevant for the early and current Mars environments. Although nitrate reduction can be coupled to anaerobic oxidation of methane (Raghoebarsing et al., 2006; Ettwig et al., 2008), the ability of NDFO strains to use C1 organic compounds as carbon sources has not been investigated. This could prove an important capability when considering the martian environment, given the as yet unexplained detections of methane in the modern atmosphere (Formisano et al., 2004; Webster et al., 2015), and should be investigated further.

Most NDFOs are heterotrophic and require an organic carbon source (Chaudhuri et al., 2001; Kappler et al., 2005; Muehe et al., 2009). Organic carbon has been reported on the martian surface and in martian meteorites (Sephton et al., 2002; Steele et al., 2012; Ming et al., 2014), which may be endogenous (Steele et al., 2012) or have been delivered into the martian crust by meteoritic input (∼2.4 × 10^5^ kg/year; Yen et al., 2006) (**Figure 5**). Sutter et al. (2016) calculated that <1% of the total carbon detected in sedimentary rocks at Gale Crater would have been sufficient to support 10^5^ cells g^-1^ sediment if present as biologically available organics in the earlier lacustrine environment, and hence could well have sustained heterotrophic NDFOs.

Although today’s martian atmosphere is oxidizing, even modest levels of volcanism over the last 3.5 billion years are likely to have produced CO_2_ at levels that contributed to periodically reducing conditions (Sholes et al., 2017), favoring NDFO by limiting abiotic iron oxidation. However, there has also been a suggestion that certain locations of the ancient surface environment (>3.5 billion years ago) were, at one point, oxidizing (Lanza et al., 2016). In practical terms, oxidizing atmospheric conditions and potential redox stratified water bodies would not preclude the viability of NDFO, but merely restrict it to anoxic sediment and water regions, as is the case on Earth.

Aside from metabolic requirements, life also needs an environment which falls within other sets of physical parameters that are conducive to life. In contrast to phototrophic iron oxidizers, NDFO could have occurred in near-surface ground waters (Straub et al., 1996), which would have protected the microorganisms even if the surface radiation environment of early Mars was as intense as it is today (Dartnell et al., 2007). In addition, cell encrustation by Fe minerals may have protected them against UV irradiation (Gauger et al., 2016). In the deep sub-surface, neutral–alkaline, Fe^2+^-rich ground waters could have persisted long after the evaporation of most surface bodies (Michalski et al., 2013), greatly extending the period across which NDFO could have been viable, possibly to the present-day.

## Biomineralization and Preservation in the Rock Record

**FIGURE 6:**
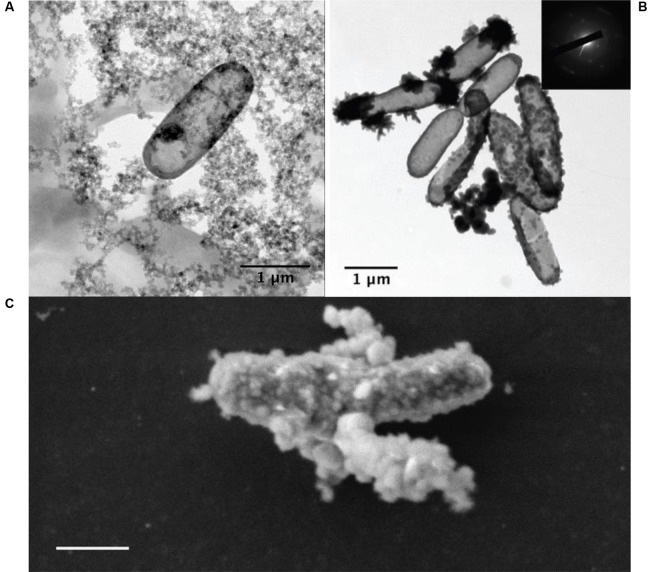
**(A)** Transmission electron microscopy (TEM) image of an iron-encrusted cell from an NDFO enrichment culture from the anoxic layer of the ferruginous Lake Pavin, France (Jennyfer Miot, personal communication). **(B)** TEM of BoFeN1 cells fully, partially, and non-encrusted with goethite (from Miot et al., 2015). **(C)** SEM of encrusted *Pseudogulbenkiania* sp. 2002 cells (from Klueglein et al., 2014) (scale bar = 500 nm).

Under Fe^2+^-rich (>5 mM) conditions, a major limiting factor for the growth of NDFO populations is the progressive encrustation of the periplasm and outer membrane by insoluble Fe^3+^ compounds (**Figure 6**), resulting in a decline in individual metabolic activity and cell death (Miot et al., 2015). Even the lithoautotrophic *Pseudogulbenkiania* sp. strain 2002 shows evidence of encrustation after batch culture (Klueglein et al., 2014) (**Figure 6C**). Although the mechanisms remain unexplained, various extracellular Fe^3+^ mineral precipitates also form as by-products of NDFO metabolism, either due to the interaction of released Fe^3+^ ions with dissolved phosphate, sulfate, and carbonate ions, or by oxidation of extracellular Fe^2+^-bearing minerals (Miot et al., 2009). Persistence of a low proportion of cells that escape encrustation ensures the viability of NDFO microorganisms at the population scale, thus accounting for their occurrence in ferruginous habitats on modern Earth (Miot et al., 2016).

The membrane-associated and extracellular mineral precipitates associated with NDFO metabolism may also present plausible biosignatures that may be detectable by future life detection missions, provided that they would persist over geological time. In particular, periplasmic encrustation leads to mineral shells that entrap protein globules and which display a constant thickness (around 40 nm) (Miot et al., 2011). The nature of the minerals has been shown to be dependent on both the local chemical composition and the pH environment. *Acidovorax* sp. strain BoFeN1, one of the best studied NDFO species, has been found to produce either lepidocrocite [γ-FeO(OH)] at pH 7 (Miot et al., 2014b) or a mixture of lepidocrocite and magnetite (Fe_3_O_4_) at pH 7.6 (Miot et al., 2014a). Likewise, changing the chemical composition of the culture medium at pH 7 results in the precipitation of either Fe^3+^ phosphates (Miot et al., 2009), goethite [α-FeO(OH)] (Kappler et al., 2005; Schädler et al., 2009), or green rust (mixed Fe^2+^/Fe^3+^ hydroxides) (Pantke et al., 2012).

It is also becoming apparent that encrustation is less likely in environments with low Fe^2+^ concentrations (50–250 μM), i.e., conditions more representative of many terrestrial NDFO sample sites (Chakraborty et al., 2011). Encrustation may occur only when solutions become highly concentrated (millimolar) with Fe^2+^ ions, as may have occurred in hydrothermal and stratified lake settings on early Mars (Hurowitz et al., 2017) or in evaporitic environments during the desiccation of the martian surface (Tosca and McLennan, 2009). Oxide-encrusted cells in both of these contexts could have been deposited and preserved during sedimentation (**Figure 4**). If deposited and lithified as macroscopic flocs or bands within an otherwise generally reducing sedimentary geological context, these oxidized mineral features may be visible in exposed strata and would serve as prime initial targets for further astrobiological investigation. Alternative mineralization processes such as pyritization (saturation and replacement of biological structures with iron sulfide) or silicification (saturation and replacement of biological structures with silica) could also contribute to non-specific morphological preservation of microbes in iron and sulfur-rich, predominantly basaltic, early martian environments. Microbial silicification has been observed on Earth *in situ* and *in vivo* around hot springs and under simulated conditions as well as in the fossil record (Toporski et al., 2002; Konhauser et al., 2004) whereas microbial pyritization is recognized only in the context of microfossils (Schieber, 2002; Wacey et al., 2013). Given the ability of microbial communities to thrive in conditions which encourage geologically rapid mineralization of biological material, these processes should not be viewed as prohibitive to microbial life on Noachian Mars, and are beneficial to the search for any traces of early life.

Formation of organo-ferric complexes has also been demonstrated to facilitate the preservation of organic molecules in soils and sediment over geological timescales on Earth (Lalonde et al., 2012), raising the possibility that encrustation of NDFO cells by Fe^3+^-bearing minerals and subsequent complexation may be beneficial to the preservation of organic biosignatures. At the same time, depending on the nature of encrusting minerals and diagenetic (T, P) conditions, Fe minerals may promote the thermal maturation of organic matter and partly erase organic biosignatures (Miot et al., 2017). It may be possible for the Mars Organics Molecule Analyzer (MOMA) mass spectrometer and Raman laser spectrometer (RLS), aboard the ESA ExoMars 2020 rover, to detect biogenic organic molecules in association with Fe^3+^ in iron-rich drill samples and laser targets, respectively (Lopez-Reyes et al., 2013; Arevalo et al., 2015). However, these instruments are not specific enough to distinguish evidence of NDFO microbes from any other potentially biological material encrusted in Fe minerals (e.g., Kish et al., 2016; Mirvaux et al., 2016).

Specific evidence of NDFO metabolism in the geological record on Earth or Mars may, however, come from isotopes. NDFOs have been shown to produce distinctive ^56^Fe/^54^Fe isotope fractionation patterns, discernible from other processes (Kappler et al., 2010). These variations may be detectable in the rock record, for example, in returned samples, using isotope ratio mass spectrometry (Anand et al., 2006; Czaja et al., 2013). The preservation of isotopic anomalies in martian sediments could provide detectable supporting evidence of NDFO on early Mars.

## Conclusion

Nitrate-dependent Fe^2+^ oxidation (NDFO) microorganisms oxidase Fe^2+^ compounds while also reducing nitrates under anaerobic, circumneutral conditions. These environments are proposed to have existed on Mars, providing the electron donors and acceptors required for NDFO metabolism. This implies that NDFO is a feasible and logical avenue for investigating hypothetical early martian life.

The discovery of nitrates establishes NDFO as a viable mechanism for hypothetical, biological iron oxidation on present-day Mars. NDFO could help to close a chemotrophic ‘loop’ of biogeochemical iron cycling on Mars, by providing a potential mechanism for iron oxidation, and allowing chemotrophic iron cycling to occur in both circumneutral ancient surface waters and deep sub-surface waters throughout martian history.

To test the validity of this hypothesis, further research should seek to determine the feasibility of NDFO metabolism under Mars simulation conditions and characterize any associated biomineralization processes. Should the suitability of NDFO to martian environments be supported by the outcomes of these experiments, future life detection missions could be optimized to seek the distinctive mineralized biosignatures of NDFO in the martian rock record.

## Author Contributions

AP was responsible for writing the manuscript with a large amount of input and revision from KO-F, VP, and SS. JM contributed to the revision, providing experience and expertise in nitrate-dependent iron oxidation and biomineralization processes. The concept for the paper was developed in discussions between AP and KO-F with VP and SS involved from the beginning.

## Conflict of Interest Statement

The authors declare that the research was conducted in the absence of any commercial or financial relationships that could be construed as a potential conflict of interest.
